# Complications of rabbit anti-thymocyte globulin induction immunosuppression in HIV-infected kidney transplant recipients

**DOI:** 10.3389/fneph.2022.1047170

**Published:** 2022-12-14

**Authors:** Ayman Al Jurdi, Esther C. Liu, Thalia Salinas, Meredith J. Aull, Michelle Lubetzky, Alexander L. Drelick, Catherine B. Small, Sandip Kapur, Choli Hartono, Thangamani Muthukumar

**Affiliations:** ^1^ Division of Nephrology, Massachusetts General Hospital, Boston, MA, United States; ^2^ Department of Pharmacy, NewYork Presbyterian Hospital-Weill Cornell Medicine, New York, NY, United States; ^3^ Division of Nephrology and Hypertension, Department of Medicine, Weill Cornell Medicine, New York, NY, United States; ^4^ Department of Transplantation Medicine, NewYork Presbyterian Hospital-Weill Cornell Medicine, New York, NY, United States; ^5^ Division of Transplant Surgery, Department of Surgery, Weill Cornell Medicine, New York, NY, United States; ^6^ Division of Infectious Diseases, Department of Medicine, Weill Cornell Medicine, New York, NY, United States; ^7^ The Rogosin Institute, New York, NY, United States

**Keywords:** HIV - human immunodeficiency virus, kidney transplanation, thymoglobulin, BK viral infection, immunosuppressant

## Abstract

**Background:**

Kidney transplantation in HIV-infected individuals with end-stage kidney disease is associated with improved survival compared to dialysis. Rabbit anti-thymocyte globulin (rATG) induction in HIV-infected kidney transplant recipients has been associated with a lower risk of acute rejection, but data on the rates of *de novo* malignancy and BK viremia in these patients is lacking.

**Methods:**

We performed a single-center retrospective cohort study of adult HIV-infected individuals who underwent kidney transplantation with rATG induction between January 2006 and December 2016. The primary outcome was the development of *de novo* malignancy. Secondary outcomes included the development of BK viremia, infections requiring hospitalization, HIV progression, biopsy-proven acute rejection, and patient and allograft survival.

**Results:**

Twenty-seven HIV-infected individuals with end-stage kidney disease received deceased (n=23) or living (n=4) donor kidney transplants. The cumulative rate of malignancy at five years was 29%, of whom 29% died because of advanced malignancy. BK viremia was detected in six participants (22%), of whom one had biopsy-proven BK virus-associated nephropathy and all of whom cleared the BK viremia. Five-year acute rejection rates, patient survival and death-censored allograft survival were 17%, 85% and 80% respectively.

**Conclusion:**

rATG induction in HIV-infected kidney transplant recipients was associated with a low risk of acute rejection, but a potentially higher risk of *de novo* malignancies and BK viremia in this cohort. Screening strategies to closely monitor for BK virus infection and malignancy post-transplantation may improve outcomes in HIV-infected kidney transplant recipients receiving rATG induction.

## Introduction

Highly active antiretroviral therapy (HAART) has had a major impact on reducing the mortality of people living with human immunodeficiency virus (HIV) with their survival now being similar to patients not infected with HIV (1). Despite the efficacy of antiretroviral therapy, the prevalence of chronic diseases such as end-stage kidney disease (ESKD) has been increasing in this population (2). People living with HIV (PWH) account for 1% of all people with ESKD in the United States (3). Disproportionately, the survival of PWH with ESKD on dialysis is lower than that of matched HIV-negative people with ESKD on dialysis (4). Kidney transplantation (KT) in PWH is associated with a 79% reduction in five-year mortality compared to dialysis and is the treatment of choice for PWH who have ESKD (5). Despite that, recent data shows that PWH who have ESKD are less likely to be referred for, evaluated for or waitlisted for KT (6).

While there may have been a reluctance to prescribe potent immunosuppressive agents in the early experience of KT in PWH given concerns about infection and malignancy risk, paradoxically, a landmark study in KT in PWH demonstrated a higher rejection rate at one-year of 31% when compared to the 12% reported in the US Scientific Registry of Transplant Recipients (7, 8). The reason for this heightened risk of rejection in already immunocompromised recipients is not fully understood. Several mechanisms were hypothesized including HIV-related modulatory effects on the immune system of the recipient resulting in expansion of alloreactive memory T cells, difficulty achieving therapeutic calcineurin inhibitor (CNI) concentrations due to drug-drug interactions with antiretroviral medications, and the reluctance to use potent lymphocyte-depleting induction agents (7, 9). Since then, studies have shown the rATG induction immunosuppression for KT in PWH is associated with a significantly reduced risk of acute rejection, which is associated with improved long-term allograft survival (10). However, data on complications of rATG induction immunosuppression for KT in PWH, especially rates of *de novo* malignancy and BK viremia, are limited.

The objective of this study was to investigate the neoplastic and infectious complications following rATG induction in PWH after KT.

## Materials and methods

This is an IRB-approved (protocol #19-04020110) retrospective cohort study of all adult HIV-infected patients who received a KT with rATG induction (1.5mg/Kg daily x4 doses) at our center between January 2006 and December 2016. Intravenous methylprednisolone was administered starting on post-operative day (POD) 0 and either tapered off by POD4 or continued at a low maintenance dose per our center’s protocol. All patients were started on maintenance immunosuppression therapy with tacrolimus and mycophenolate. Opportunistic infection prophylaxis was initiated post-transplant per our center’s protocol for oral candidiasis, pneumocystis pneumonia, and cytomegalovirus (CMV) infection using clotrimazole, sulfamethoxazole-trimethoprim, and valganciclovir or acyclovir, respectively. There were no restrictions on the use of antiretroviral medications. Only for-indication biopsies were performed during follow-up. No protocol biopsies were performed. Details of post-transplant immunosuppression, infection prophylaxis and monitoring are described in **Supplementary Materials and Methods**.

The primary outcome was the development of *de novo* malignancy within five years after transplantation. Secondary outcomes included the development of BK viremia, infection requiring hospitalization, HIV progression, biopsy-proven acute rejection within five years of transplantation, and patient and graft survival at one and five years. Graft failure was defined as return to maintenance dialysis. We used Kaplan-Meier analysis to estimate patient and allograft survival, time to first episode of rejection, and infection requiring hospitalization. Patients who did not reach their primary outcomes were censored at their last follow up. We used GraphPad Prism version 8.4.3 for statistical analyses. The study was conducted in compliance with the Declaration of Helsinki and all data are reported in compliance with STROBE guidelines. The clinical and research activities being reported are consistent with the Principles of the Declaration of Istanbul as outlined in the ‘Declaration of Istanbul on Organ Trafficking and Transplant Tourism’.

## Results

### Characteristics of kidney allograft donors and recipients

Between January 1, 2006, and December 31, 2016, 27 PWH received deceased (n=23) and living (n=4) KTs and induction immunosuppression with rATG at our center. **Table 1** describes the donor and recipient characteristics. Twenty-six patients were on hemodialysis and one patient was on peritoneal dialysis prior to transplantation. All patients were started on maintenance immunosuppression with tacrolimus and mycophenolate. One patient was switched from tacrolimus to cyclosporine prior to discharge because of tacrolimus nephrotoxicity. The remaining patients were discharged on tacrolimus. Five patients (18%) were discharged on corticosteroids but only three (11%) were maintained on corticosteroids long term. Patients were followed for a median of 63 months.

**Table 1:** Donor and recipient characteristics.

Characteristics	N
Donor (N=27)	
Age, median (IQR)	46 (24-53)
Female, n (%)	15 (56)
Black, n (%)	3 (11)
Deceased, n (%)	23 (85)
Kidney donor profile index, median, % (IQR)	54 (38-75)
Recipient (N=27)	
Age, median (IQR)	53 (39-59)
Female, n (%)	9 (33)
Black, n (%)	18 (67)
Body mass index, Kg/m^2^, median (IQR)	25 (22-28)
Etiology of end-stage kidney disease, n (%)	
Presumed HIV-associated nephropathy Presumed diabetic nephropathy Biopsy-confirmed HIV-associated nephropathy Other	18 (67) 2 (7) 2 (7) 5 (19)
Pre-transplant diabetes mellitus, n (%)	4 (15)
Time on dialysis, months, median (IQR)	90 (57-114)
Previous kidney transplant, n (%)	1 (4)
Duration of HIV infection, months, median (IQR)	206 (131-270)
Anti-retroviral medications, n (%)	
Nucleoside reverse transcriptase inhibitors Non-nucleoside reverse transcriptase inhibitors Protease inhibitors Integrase inhibitors	25 (93) 13 (50) 15 (56) 11 (41)
Previous opportunistic infections, n (%) *Pneumocystis jiroveci* pneumonia Central nervous system toxoplasmosis Cytomegalovirus retinitis Oropharyngeal candidiasis	6 (22) 3 (11) 1 (4) 1 (4) 1 (4)
CD4 count, cells/mm^3^, median (IQR)	478 (407-520)
Patients with pre-transplant malignancy, n (%) Anal squamous cell carcinoma Anal intraepithelial neoplasia Prostate cancer Papillary thyroid carcinoma* Kaposi’s sarcoma* Cervical low-grade squamous intraepithelial lesion	7 (26) 2 (7) 2 (7) 2 (7)*^a^* 1 (4) *^a^* 1 (4)*^a^* 1 (4)
Hepatitis B virus infection, n (%)	2 (7)
Hepatitis C virus infection, n (%)	4 (15)
Cold ischemia time, hours, median (IQR)	27 (13-33)
Complement dependent cytotoxicity crossmatch, data available, n (%) T-cell positive B-cell positive	27 (100) 0 (0) 0 (0)
Luminex platform donor specific antibody, data available, n (%) Positive*^b^*	26 (96) 3 (12)
Human leukocyte antigen ABDR mismatches, n (%)	
0 3 4 5 6	2 (7) 2 (7) 4 (15) 8 (30) 11 (41)
Early corticosteroid withdrawal, n (%)	24 (89)

^a^One patient had both prostate cancer and papillary thyroid carcinoma. Another patient had both prostate cancer and Kaposi’s sarcoma.

^b^Circulating antibodies against donor IgG were measured in the serum of transplant recipients on a Luminex platform. A mean fluorescence intensity value of 2000 or more was considered as positive.

### *De novo* malignancy

**Figure 1:**
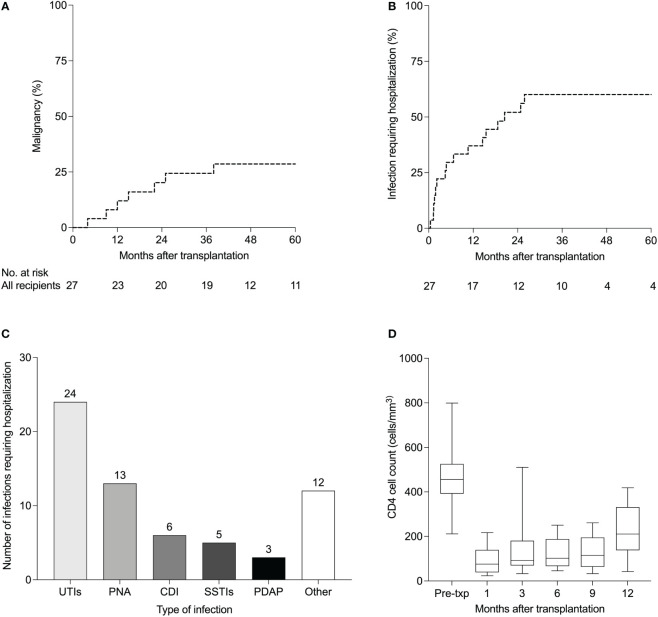
Complications of anti-thymocyte globulin induction in HIV-infected kidney transplant recipients (n=27). **(A)** Kaplan-Meier estimates of *de novo* malignancies and **(B)** infections requiring hospitalization after kidney transplantation. **(C)** Details of infections requiring hospitalization **(B)** in 27 HIV-infected kidney transplant recipients managed with. Other infections included cholecystitis (n=2), bacteremia (n=2), implantable cardioverter-defibrillator infection (n=1), *Candida glabrata* fungemia (n=1), pulmonary tuberculosis (n=1), *Pneumocystis jirovecii* pneumonia (n=1), Rhinovirus (n=1), Cytomegalovirus colitis (n=1), liver abscess (n=1) and Shiga toxin-producing *E coli* (n=1). **(D)** CD4 cell counts (cells/mm^3^) after kidney transplantation. Boxplots show the 10^th^, 25^th^, 50^th^, 75^th^, and 90^th^ percentiles. UTIs, urinary tract infections; PNA, pneumonia; CDI, *Clostridium difficile* infection; SSTIs, skin and soft tissue infections; PDAP, peritoneal dialysis-associated peritonitis.

Seven patients developed seven *de novo* malignancies during the first five years after KT (five-year Kaplan-Meier estimate 29%, **Figure 1A**). Three of the seven patients who developed *de novo* malignancies had a malignancy prior to transplantation. Five of the seven patients remain alive with either no progression or no recurrence of malignancy, while two died from advanced malignancy. Details of the treatment and outcomes of patients who developed post-transplant malignancies are summarized in **Table 2**.

**Table 2:** Outcomes of HIV-infected individuals with kidney transplantation who developed malignancies within five years of transplantation.

No.	Malignancy	Diagnosis (Months after transplant)	Treatment	Outcome	Last follow-up (Months from transplant)	Pretransplant malignancy
1	Skin BCC	4	Mohs surgery	Alive with no evidence of recurrence	90	Kaposi’s sarcoma and prostate cancer
2	Low-grade AIN	38	Observation	Alive with no progression of low-grade AIN	78	None
3	Non-small cell lung (metastatic)	25	Systemic chemotherapy	Died three months after diagnosis from infection and progression of disease	27	None
4	Skin SCC in situ	9	Mohs surgery	Alive with no evidence of recurrence	65	None
5	High-grade AIN	22	Surgical excision	Alive with no evidence of recurrence	34	Prostate and papillary thyroid
6	Tonsillar SCC (metastatic)	15	Radiation and systemic chemotherapy	Died five months after diagnosis from progression of disease	20	Anal cancer
7	Low-grade AIN	12	Observation	Alive with no progression of AIN	29	None

AIN, anal intraepithelial neoplasia; BCC, basal cell carcinoma; SCC, squamous cell carcinoma.

### Infections

At 12 months, 37% of patients developed infections requiring hospitalization. Five-year Kaplan-Meier estimate for infections requiring hospitalization was 60% (**Figure 1B**). The most common infections resulting in hospitalization were urinary tract infections, pneumonias, and *Clostridium difficile* infections (**Figure 1C**). In terms of viral infections after transplantation, BK viremia developed in six (22%) patients at a median of 7 months (IQR 5-14) post-transplantation, of whom one had biopsy-proven BK virus-associated nephropathy. BK viremia resolved in all patients at a median of 5.5 months (IQR 3.9-29.4). Details of BK viremia diagnosis and outcomes are summarized in **Supplementary Table S1**. Epstein-Barr virus (EBV) and CMV viremia developed in two and five patients respectively. Of the five patients who developed CMV viremia, the CMV serostatus was D+/R+ in three patients and D+/R- in two patients. Two patients developed Hepatitis B (HBV) viremia, both of whom had pre-transplant HBV infection. Five patients developed Hepatitis C (HCV) viremia, four of whom had pre-transplant HCV infection. One patient developed new HCV infection 79 months post-transplantation for unclear reasons as he denied intravenous or intranasal drug use, high-risk sexual behavior, body piercings or tattoos.

### Antiretroviral regimens and HIV Progression

Composition of antiretroviral regimens at the time of transplant is shown in **Table 1**. Antiretroviral therapy was re-started on the day of transplant in 32%, on POD1 in 48% and on POD2 in the remaining 19% of patients. Immediately after transplantation, 13 patients were on a ritonavir-based regimen while the remaining patients were on non-ritonavir-based regimens. At their last follow up visit, seven patients remained on a ritonavir-based regimen while the remaining patients were on non-ritonavir-based regimens. Median CD4 cell counts at 1, 3, 6, 9 and 12 months after transplantation are shown in **Figure 1D**. Nadir CD4 cell counts did not differ between those who did vs did not develop *de novo* malignancies (*P* = 0.534, **Figure S1A**) and did not differ between those who did vs did not develop BK viremia (*P* = 0.801, **Figure S1B**). At 12 months, only four patients had detectable HIV viral loads. Three of the four patients had low level HIV viremia (<200 copies/mL) and were monitored without changes in their antiretroviral regimens. Their viral loads subsequently became undetectable. The fourth patient had a viral load of 47,520 copies/mL in the setting of antiretroviral medication non-compliance. He was re-started on his previous antiretroviral regimen and his viral load subsequently became undetectable.

### Patient and allograft survival

**Figure 2:**
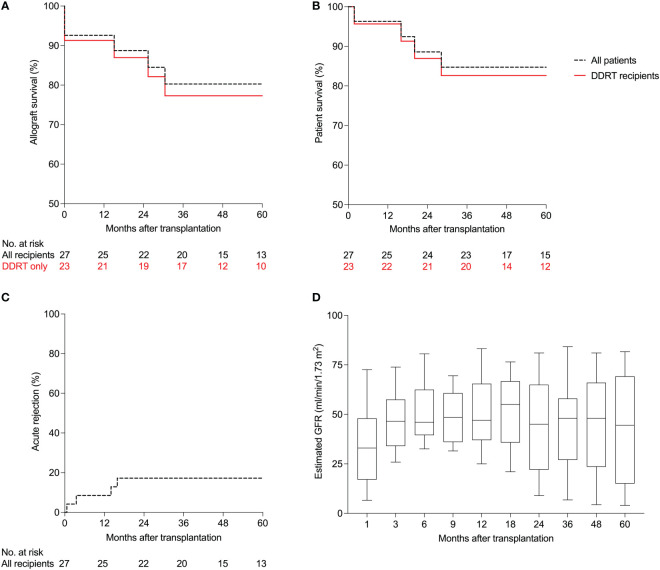
Patient and allograft outcomes of HIV-infected kidney transplant recipients managed with anti-thymocyte globulin induction (n=27). **(A)** Kaplan-Meier estimates of allograft survival, **(B)** patient survival, **(C)** and acute rejection. **(D)** Estimated glomerular filtration rates (GFR) using the 4-parameter Modification of Diet in Renal Disease (MDRD) formula. Boxplots show the 10^th^, 25^th^, 50^th^, 75^th^, and 90^th^ percentiles. DDRT, deceased donor renal transplant.

At 12 months after transplantation, patient survival was 96% and death-censored allograft survival was 93%. One patient died 44 days post-transplant from septic shock secondary to pneumonia. Two patients did not recover renal function after transplant (primary nonfunction) and both patients underwent kidney biopsies. One patient had tubular injury that did not improve, and the other patient had mixed acute rejection that did not improve with treatment. Both patients had to resume maintenance hemodialysis. Five-year Kaplan-Meier survival estimate was 80% for allograft survival (**Figure 2A**) and 85% for patient survival (**Figure 2B**). In recipients of deceased donor renal transplants (DDRT), allograft and patient survival were 91% and 96% at one year, and 77% and 83% for five-year Kaplan-Meier survival estimates. There were no patient deaths or allograft loss in recipients of living donor transplants during follow-up. **Supplementary Table S2** summarizes post-transplant outcomes of the cohort.

### Acute rejection

Within 12 months, two patients (7%) developed acute rejection. One patient (discussed above) developed a mixed acute rejection (acute T-cell mediated and vascular rejection, Banff grade IIB, and acute antibody-mediated rejection) at three weeks post transplantation. The patient was treated with pulse methylprednisolone, intravenous immunoglobulins and plasmapheresis but did not recover kidney function. The patient was re-started on hemodialysis and underwent a transplant nephrectomy.

The second patient developed acute T-cell mediated rejection (Banff grade IB) three months post-transplantation with an increase in serum creatinine from 1.78 to 3.25 mg/dL. The patient was treated with pulse intravenous methylprednisolone followed by an oral prednisone taper with improvement in allograft function to a serum creatinine of 1.76 mg/dL two weeks after treatment. At five years, the Kaplan-Meier estimate for acute rejection was 17% for the entire cohort (**Figure 2C**). In the subgroup of patients managed with an early corticosteroid withdrawal protocol, rates of acute rejection were 8% at one year and 17% at five years.

### Allograft function, tacrolimus levels and immune sensitization

At the time of transplant, none of the patients had a positive T or B crossmatch by complement-dependent cytotoxicity assay. Results of the flow cytometric crossmatch were available in five patients; all were negative for T and B cell crossmatch. Luminex platform test to detect circulating IgG directed against donor human leukocyte antigen (HLA) using single antigen beads was done in 26 of the 27 (96%) recipients. Three were positive at the time of transplant based on our threshold of 2000 mean fluorescence intensity value. After transplant, these three recipients tested negative for donor-specific antibodies (DSAs) and at the last follow up (median follow up 63 months) had no DSAs, stable allograft function and no proteinuria. Three patients developed *de novo* DSAs after transplantation and at their last follow up (median follow up 27 months) had stable allograft function and no proteinuria. Glomerular filtration rates estimated using the 4-parameter Modification of Diet in Renal Disease (MDRD) formula (eGFR), at 1, 3, 6, 9, 12, 18, 24, 36, 48 and 60 months after transplantation are shown in **Figure 2D**. At 36 months, only three patients had more than one gram of proteinuria. Tacrolimus trough levels in the first 36 months after transplantation for the entire cohort are shown in **Supplementary Figure S2A**. Tacrolimus levels stratified by ritonavir-based vs non-ritonavir-based regimen are shown in **Supplementary Figure S2B** and **S2C**, respectively.

## Discussion

Kidney transplantation for HIV-infected patients with ESKD is associated with improved survival compared to dialysis (5). The optimal induction immunosuppression regimen for HIV patients who are immunocompromised at baseline has not been established. Induction with rATG reduces the risk of acute rejection in this high-risk group (10). However, it is important to balance the risks of rejection, infection, and malignancy when choosing induction immunosuppression, especially since HIV infection itself (11–13), kidney transplantation (14), and rATG induction immunosuppression (15) are each associated with a higher risk of certain malignancies. Since the associated risk of *de novo* malignancy and BK viremia has not been studied thoroughly yet in this patient population, we aimed to evaluate these risks in this study.

An important finding in our study was that the incidence of malignancy after transplant was high (29% within five years) and about half of these patients had pre-transplant malignancies. Five of the seven patients who developed post-transplant malignancies remain alive with no evidence of recurrence or progression of their malignancies. Two of the seven malignancies were metastatic, resulting in death. While this rate of malignancy is high, it is difficult to interpret as there is little data on the risk of post-transplant malignancies in this group of patients and its impact on mortality (16). Nissen et al. reported that 13 out of 150 PWH (9%) developed 14 post-transplant malignancies and three cancer-related deaths at a shorter median follow-up of 3.5-year after KT (17). In comparison, a study of 240 non-HIV-infected DDRT recipients who received rATG induction, eight patients (3%) developed malignancies in the first year (18), compared to three (11%) in the first year in our study. We hypothesize that the higher risk of malignancy reported in our study may be due in part to rATG induction and to the fact that we routinely screen HIV-infected patients for anal intra-epithelial and invasive malignancies, which accounted for three of the seven cases in our study. This is supported by a cross-sectional study that showed that on screening, anal cytological abnormalities were present in 12% in HIV-negative KT recipients (19) and the risk is likely higher in HIV-infected individuals (20). Another possible contributor may be the high percentage of patients with pre-transplant malignancies in our group, which may be associated with a higher risk of malignancy after transplant (21). However, the proportion of patients with pre-transplant malignancy has not been reported in other studies to be able to make comparisons. Therefore, until more data becomes available, rATG induction should be used with caution in HIV-infected patients at high risk for post-transplant malignancies, such as those with a history of pre-transplant malignancies. HIV-infected KTRs who receive rATG induction should likely undergo careful monitoring for malignancy after KT to increase the chances of finding and treating earlier-stage malignant or pre-malignant lesions.

The median CD4 count markedly dropped below 200 cells per mm^3^ soon after rATG induction in our transplant recipients. Contrary to the tenet of preserving the CD4 count when managing non-transplant HIV-infected patients, it is alarming that the CD4 count did not recover to 200 cells per mm^3^ until almost 12 months after induction therapy. Despite that, only one patient developed an opportunistic infection during the first 12 months post-transplant (*Pneumocystis jirovecii* pneumonia at 4.5 months) and there were no cases of progressive multifocal leukoencephalopathy. BK viremia, however, developed in 22% of patients, which is higher than the 11.5-12.6% reported in non-HIV-infected kidney transplant recipients (22, 23). However, biopsy-proven BK virus nephropathy was diagnosed in only one patient, and BK viremia cleared in all patients. BK viremia and nephropathy have been reported previously in HIV-infected KT recipients (24, 25), but the incidence in this group is not known. Potential explanations for the higher incidence of BK viremia in our cohort include HIV infection and the universal use of rATG for induction immunosuppression, which has been associated with a higher risk of BK viremia (26, 27). However, in our cohort, BK virus infection did not result in allograft loss in any of the patients. Therefore, the potentially higher risk of BK viremia in HIV-infected KTRs who receive of rATG for induction immunosuppression should prompt that these patients have frequent monitoring for the development of BK viremia, but should not discourage the use of rATG in this population.

The most common infections observed were urinary tract infections, similar to what has been reported in previous studies of HIV-infected KT recipients (28, 29). Infections requiring hospitalization occurred in 37% of patients in the first year. Previous studies have reported similar rates of infections with rATG use. In a study of 240 non-HIV deceased DDRT recipients who received rATG induction, 43% of patients developed significant infections in the first year, although the mean rATG dose reported (8.8 mg/Kg) was higher than what is typically used now (18). Stock et al. reported a 38% rate of infections requiring hospitalization in HIV-infected KT recipients at a median follow up of 1.7 years. Only 32% of their patients had received rATG induction and the rate of infections was twice as high in patients who received rATG induction compared to those who did not (7). Kucirka et al. reviewed induction in HIV-infected KT recipients using registry data and reported a 52.8% risk of infection in patients who received rATG induction in the first year after KT, which was similar to patients who received induction with an anti-CD25 antibodies (52.5%) or no induction (55.7%) (29). While there is some discrepancy between the studies’ findings, taken together they suggest that rATG induction is likely not associated with a significantly higher risk of serious infections in HIV-infected KT recipients. A potential explanation for that is that rATG induction reduces the risk of acute rejection, which would require additional immunosuppression, therefore resulting in an overall similar cumulative “dose” of immunosuppression and not increasing the overall risk of post-transplant infections (29).

With regards to patient outcomes, we observed similar one and five-year patient survival to previously reported HIV-infected KT recipients (7, 16, 30). Rates of kidney allograft survival in HIV-infected recipients have varied widely in previous studies, with one-year survival rates ranging between 75% and 100% (7, 28, 31–34). The one-year death-censored allograft survival in our study was 93%, which is higher than previously reported numbers in HIV-infected recipients in older studies and closer to more recently reported ones (7, 28, 30, 31). Five year death-censored allograft survival in our study was 80%, which is also higher than what has been reported from older cohorts (30%) and comparable to more recent cohorts (60-87%) (28, 30, 32, 35). In our study, there was a 7% incidence of acute rejection at one year, which is lower than most previously published data in HIV and similar to non-HIV infected KT recipients (7, 8, 16, 30). One published study had similar findings to ours with regards to allograft rejection rates in HIV-infected KTRs, which was 8% at one year and 22% at three years in 27 HIV-infected KTRs. In this study, all HIV-infected KTRs received high doses of ATG induction (five to seven doses of 1.5-2.0 mg/Kg) and all were maintained on tacrolimus, mycophenolate and prednisone immunosuppression (28).

Our data also adds evidence that HIV-infected KT recipients receiving rATG induction immunosuppression have similar five-year patient survival when compared to non-HIV KT recipients from the Scientific Registry of Transplant Recipients (SRTR) database (83% vs 87% at five years for DDRTs). Rates of death-censored allograft failure while similar at one year between our study and SRTR data (9% vs 5% for DDRTs) but were higher in our HIV-infected patient cohort at five years (23% vs 12% for DDRTs). Rates of acute rejection were also similar at one-year (7% vs 8% overall). The five-year rate of acute rejection in our cohort was 17% but was not specified in the SRTR data (8). While steroid maintenance regimens have been associated with a lower risk of acute rejection but similar death-censored allograft and patient survival in HIV-infected KTRs (35), due to the small number of KTRs on steroid maintenance regimens in our study, we were not able to compare differences between those on steroid maintenance and steroid withdrawal regimens.

Our study has several limitations including its retrospective single-center design, lack of a matched control group managed without rATG induction and the small sample size of our cohort. Despite that, our study demonstrates the efficacy of rATG induction immunosuppression in high-risk HIV-infected KT recipients with similar long-term patient survival and one-year acute rejection rates when compared to non-HIV infected KT recipients. Rates of death-censored allograft survival are similar to non-HIV infected KT recipients at one year but are lower at five years. While rates of BK viremia and *de novo* malignancy appear higher in this group of patients, this needs to be weighed against the benefit of lower risk of acute rejection in this population. Therefore, our findings should not discourage the use of rATG induction immunosuppression in this population given its clear benefits in lowering the risk of acute rejection. Rather, these patients should be considered for more frequent screening for malignancy and BK viremia to mitigate these risks. Prospective studies with larger cohorts are needed to investigate both the incidence and prevention strategies for BK virus infection and *de novo* malignancy in HIV-infected KT recipients receiving rATG induction.

## Data availability statement

The original contributions presented in the study are included in the article/**Supplementary Material**. Further inquiries can be directed to the corresponding author.

## Ethics statement

The studies involving human participants were reviewed and approved by Weill Cornell Medicine IRB protocol #19-04020110. Written informed consent for participation was not required for this study in accordance with the national legislation and the institutional requirements.

## Author contributions

AJ, EL, AD, CS, SK, CH, and TM designed the research project. AJ, EL, TS, AD, and TM collected the data. AJ, EL, MA, ML, AD, CS, CH, and TM analyzed the data. AJ, EL, CH, and TM wrote the manuscript and reviewed the final version. All authors contributed to the article and approved the submitted version.
