# Supplementary material for: Complications of rabbit anti-thymocyte globulin induction immunosuppression in HIV-infected kidney transplant recipients
Source: Front Nephrol. 2022 Dec 14;2:1047170. doi: 10.3389/fneph.2022.1047170 (PMC10479633; doi:10.3389/fneph.2022.1047170)
Supplement: Supplementary file 1 [file DataSheet_1.docx]

**Supplemental table of contents:**

| - Supplementary materials and methods | Page 1 |
| --- | --- |
| - Supplementary Figure S1 | Page 2 |
| - Supplementary Figure S2 | Page 3 |
| - Supplementary Tables S1-S2 | Pages 4-5 |

**Supplementary materials and methods**

Immunosuppression protocol at our center:

- Induction glucocorticoids: IV methylprednisolone 500 mg on POD0, 250 mg no POD1, 125 mg on POD2, and 60mg on POD3 and POD4. Patients who are deemed to be high immunologic risk (e.g., having donor-specific anti-HLA antibodies with mean fluorescent intensity >3,000) are continued on prednisone 5 mg daily long-term (i.e., steroid maintenance). Patients who are deemed to be at low immunologic risk do not receive long-term steroids (i.e., early steroid withdrawal).
- Tacrolimus: 2 mg twice daily then adjusted to achieve levels of 8-10 ng/mL
- Mycophenolate mofetil: 1000 mg once pre-op and then 1000 mg twice daily until POD4. POD5 onwards: starting dose is 500 mg twice daily for all patients except for African American, highly sensitized, or basiliximab induction (not included in this study) who may receive up to 1000 mg twice daily.

Prophylaxis protocol:

- *Pneumocystis jirovecii*: TMP-SMX single strength tablet for one year.
- Fungal: clotrimazole 10 mg troche twice daily for 1-3 months.
- CMV:
  - Low-risk (D-/R-): oral acyclovir for 3 months
  - Moderate or high risk (D+/R+, D-/R+, D+/R-): oral valganciclovir for 6 months

Monitoring protocol:

- BK viremia: monitoring is typically performed at months 1, 3, 6, 9, 12, 18, 24 and 36 after kidney transplantation.
- Proteinuria: urinalysis monitoring for proteinuria was performed at months 1, 3, 6, 9, 12, 18, 24 and 36 after kidney transplantation. Patients with positive results on urinalysis had quantification using either a spot urine protein-creatinine (UPCR) or albumin-to-creatinine ratio (UACR) or a 24-hour urine collection for protein. Some providers also routinely monitor UPCR or UACR regardless of urinalysis results.
- Cancer screening: in addition to standard-of-care screening recommended by USPSTF, HIV-infected KTRs at our center underwent screening for HPV-related cancers by oropharyngeal examination and anal pap smears during post-transplant follow-up visits.

**Supplementary figures**

**
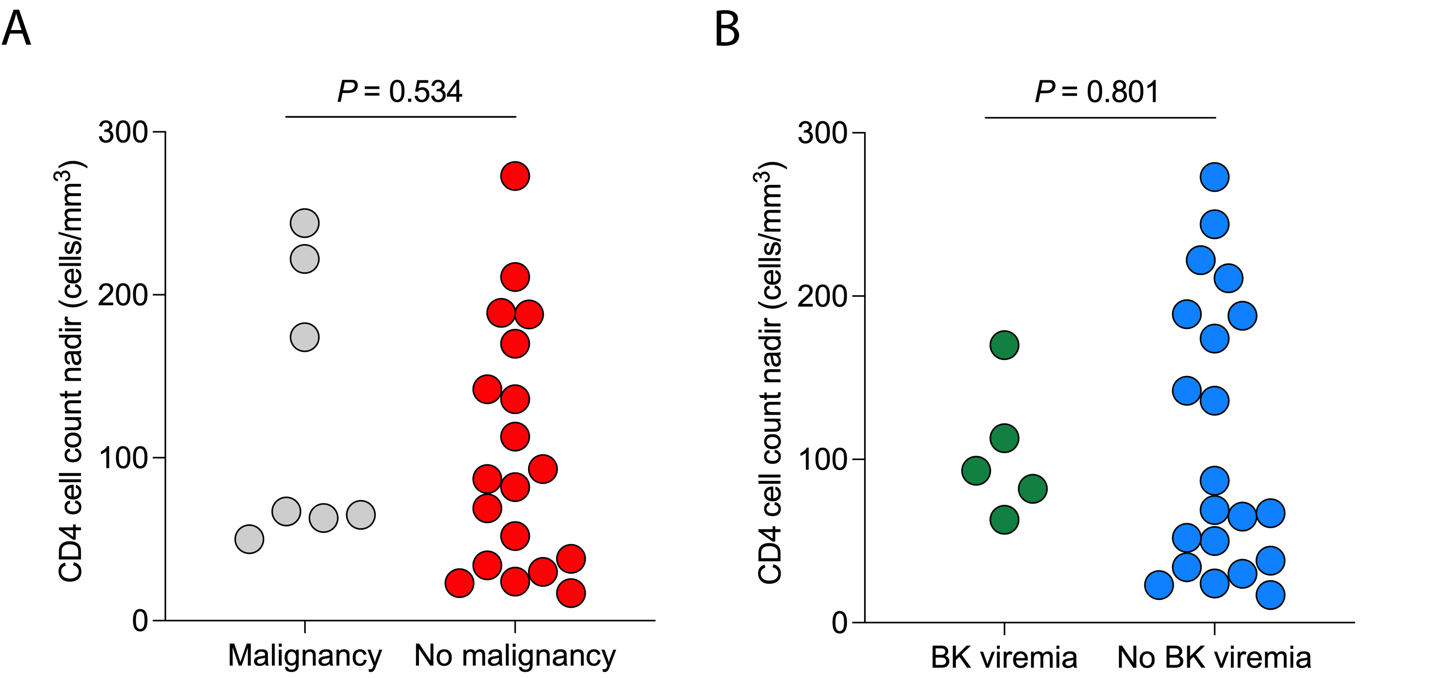
**

**Supplementary Figure S1**. Nadir CD4 cell counts in the first year in HIV-infected kidney transplant recipients stratified by the development of post-transplant **(A)** *de novo* malignancy and **(B)** BK viremia. (A,B) Statistics by Mann Whitney U test.


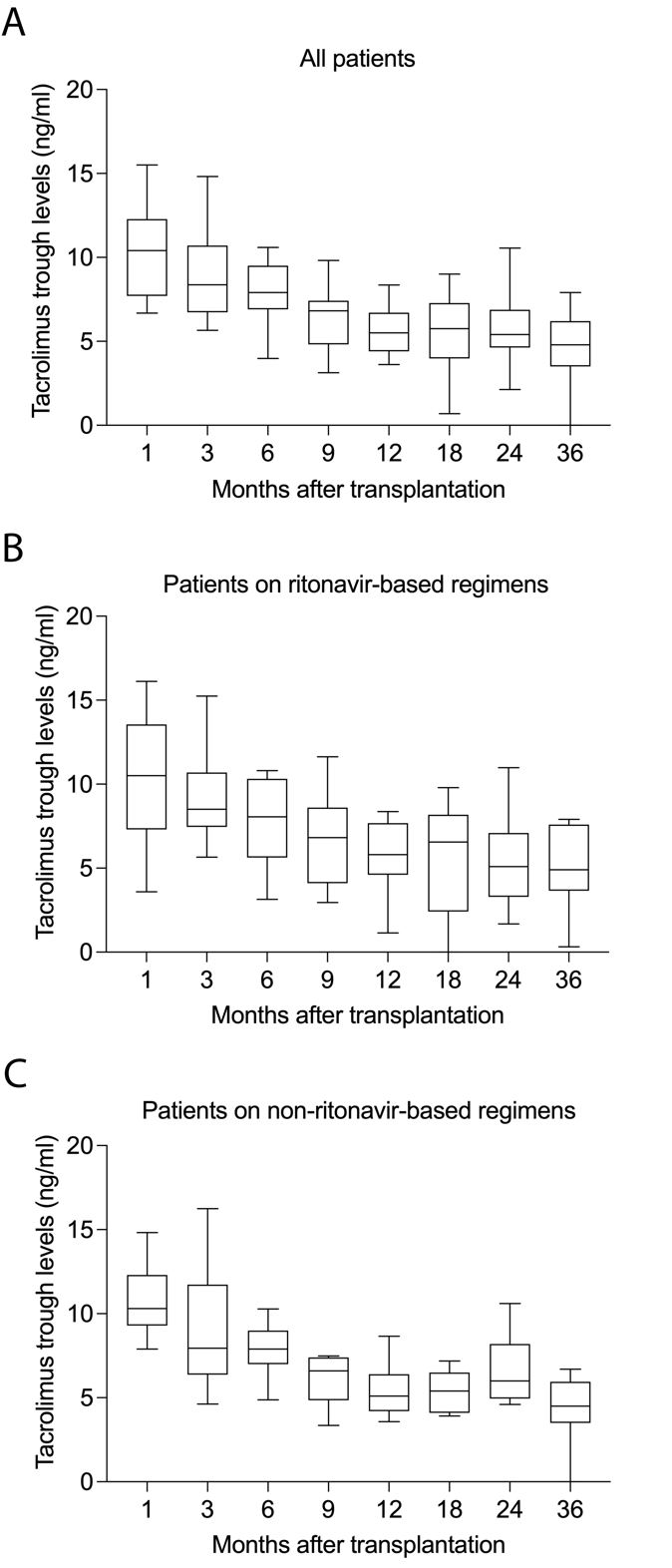


**Supplementary Figure S2**. Tacrolimus levels after kidney transplantation in HIV-infected transplant recipients in **(A)** the entire cohort (n=27) and stratified by those on **(B)** ritonavir-based (n=13) vs **(C)** non-ritonavir-based regimens (n=14). Boxplots show the 10th, 25th, 50th, 75th, and 90th percentiles.

**Supplementary tables**

**Table S1. BK viremia in HIV-infected individuals within five years of kidney transplantation.**

| **No.** | **Months since transplant** | **Peak viral load (copies/mL)** | **Biopsy-proven BKVN?** | **Treatment** | **Outcome** |
| --- | --- | --- | --- | --- | --- |
| 1 | 16 | 5,432 | Biopsy without BKVN | Reduction in IS | Resolution of BK viremia |
| 2 | 6 | 500 | Not biopsied | Reduction in IS | Resolution of BK viremia |
| 3 | 8 | 133,415 | Yes | Reduction in IS | Resolution of BK viremia |
| 4 | 4 | 68,194 | Biopsy without BKVN | Reduction in IS | Resolution of BK viremia |
| 5 | 25 | 110,000,000 | Biopsied at outside hospital where BKV stain was not done | Reduction in IS + IVIg + leflunomide | Resolution of BK viremia |
| 6 | 4 | 611 | Not biopsied | Reduction in IS | Resolution of BK viremia |

BKVN: BK virus-associated nephropathy. IVIg: intravenous immunoglobulin. IS: immunosuppression.

**Table S2. Post-transplant allograft and patient outcomes**

| **Outcomes** | **N (%)** |
| --- | --- |
| Delayed graft function | 13 (48) |
| One-year patient survival | 26 (96%) |
| One-year death-censored graft survival | 25 (93%) |
| One-year biopsy-proven acute rejection | 2 (7%) |
| Five-year patient survival Kaplan-Meier estimate | 85% |
| Five-year death-censored graft survival Kaplan-Meier estimate | 80% |
